# Body adiposity markers and insulin resistance in patients with type 1 diabetes

**DOI:** 10.20945/2359-3997000000599

**Published:** 2023-02-07

**Authors:** Camila Lemos Marques, Mileni Vanti Beretta, Raquel Eccel Prates, Jussara Carnevale de Almeida, Ticiana da Costa Rodrigues

**Affiliations:** 1 Universidade Federal do Rio Grande do Sul Programa de Pós-graduação em Endocrinologia Porto Alegre RS Brasil Universidade Federal do Rio Grande do Sul, Programa de Pós-graduação em Endocrinologia, Porto Alegre, RS, Brasil; 2 Universidade Federal do Rio Grande do Sul Departamento de Nutrição Faculdade de Medicina Porto Alegre RS Brasil Universidade Federal do Rio Grande do Sul, Departamento de Nutrição, Faculdade de Medicina, Porto Alegre, RS, Brasil; 3 Universidade Federal do Rio Grande do Sul Departamento de Clínica Médica Faculdade de Medicina Porto Alegre RS Brasil Universidade Federal do Rio Grande do Sul, Departamento de Clínica Médica, Faculdade de Medicina, Porto Alegre, RS, Brasil

**Keywords:** Insulin resistance, type 1 diabetes, intra-abdominal fat, body adiposity

## Abstract

**Objectives:**

Body composition changes are associated with adverse effects such as increased insulin resistance (IR) in individuals with diabetes mellitus. This study aims to evaluate the association between different body adiposity markers and IR in adults with type 1 diabetes (T1D).

**Subjects and methods:**

The cross-sectional study included outpatient adults with T1D from a university public hospital in southern Brazil. The body adiposity markers studied were waist circumference (WC), waist-height ratio (WHtR), body mass index (BMI), conicity index (CI), lipid accumulation product (LAP) and body adiposity index (BAI). IR was calculated using an Estimated Glucose Disposal Rate (EGDR) equation (analyzed in tertiles), considering an inverse relation between EGDR and IR. Poisson regression models were used to estimate the odds ratio (OR) and 95% CIs of association of adiposity markers with IR.

**Results:**

A total of 128 patients were enrolled (51% women), with a median EGDR of 7.2 (4.4-8.7) mg.kg^−1^.min^−1^. EGDR was negatively correlated with WC (*r* = −0.36, p < 0.01), WHtR (*r* = −0.39, p < 0.01), CI (*r* = −0.44, p < 0.01), LAP (*r* = −0.41, p < 0.01) and BMI (*r* = −0.24, p < 0.01). After regression analyses, WC (OR = 2.07; CIs: 1.12-3.337; p = 0.003), WHtR (OR = 2.77; CIs: 1.59-4.79; p < 0.001), CI (OR = 2.59; CIs: 1.43-4.66; p = 0.002), LAP (OR = 2.27; CIs: 1.25-4.11; p = 0.007) and BMI (OR = 1.78; CIs: 1.09-2.91; p = 0.019) remained associated with IR.

**Conclusions:**

The authors suggest using the studied adiposity markers as a routine since they were shown to be suitable parameters in association with IR.

## INTRODUCTION

Obesity, especially abdominal adiposity, is related to clinical and metabolic complications such as dyslipidemia, hypertension (1), type 2 diabetes (T2D) and insulin resistance (IR) (2). IR has also been attributed to type 1 diabetes (T1D) (3), and it increases the risk for micro- and macrovascular complications (4). Subjects with IR have a raised risk of cardiovascular disease development (5,6), and a high risk of mortality (6). Cardiovascular disease is the most common cause of death and disability among individuals with diabetes, especially in subjects showing inadequate control of diabetes (6).

The prevalence of overweight is spreading among individuals with T1D (7); this increase in body fat is associated with IR (8) and consequently with cardiovascular risk (9) and kidney disease (10). Insulin sensitivity decreases physiologically during puberty, with insulin requirements being increased by up to 30% in adolescent individuals with T1D (11). Furthermore, weight gain and high daily insulin requirements for long periods are associated with adverse changes such as IR and risk of cardiovascular disease (12).

The evaluation of body adiposity is essential due to the known association between abdominal obesity and IR (8). Methods that assess body composition, such as dual-energy X-ray absorptiometry (DEXA) and computed tomography, are more precise when identifying abdominal obesity (13), but their use in clinical practice is limited due to their high cost and complexity. However, anthropometric measurements and their related indexes are non-invasive, easy to apply and low cost; for instance, measurements such as waist circumference (WC), waist-hip ratio (WHR), waist-height ratio (WHtR), body mass index (BMI), conicity index (CI), lipid accumulation product (LAP), and body adiposity index (BAI) can be considered body adiposity markers by the evaluation of adipose tissue distribution (14-18). These measurements have been studied in association with cardiometabolic risk in T2D patients and healthy individuals (16-18); however, these measurements associated with diabetes complications in subjects with T1D are still poorly studied.

The identification of excess body adiposity ensures early intervention and possible impact to prevent chronic complications of diabetes. Since the best marker of body fat for individuals with T1D is not clearly known, especially related to IR, this study aims to evaluate different body adiposity markers in adults with T1D and the possible association between these markers and IR.

## SUBJECTS AND METHODS

### Study population

A cross-sectional study with outpatient T1D adults was carried out in the Endocrinology Division at a public hospital in southern Brazil. Trained individuals collected data from 2008 to 2013 by convenience sampling. T1D was defined by onset before 40 years of age, the presence of ketonuria or ketonemia at the time of diagnosis, and dependence on insulin therapy to sustain life. The inclusion criteria were age between 18-59 years and a diagnosis of T1D for more than five years. Exclusion criteria were hemodialysis and/or decompensated heart failure.

This study was conducted in accordance with the Declaration of Helsinki and was approved by the Research Ethics Committee (CAAE:22905313.7.0000.5327). Written informed consent was obtained from all patients.

### Laboratorial and clinical evaluation

Blood tests were performed after twelve hours of fasting and analyzed at the Pathology Laboratory of the hospital. The evaluation of fasting plasma glucose was performed by the enzymatic colorimetric glucose-peroxidase method (Biodiagnóstica^®^, Brazil), glycated hemoglobin (HbA1c) was analyzed using high performance liquid chromatography (Merck-Hitachi 9100; Merck^®^, Darmstadt, Germany), serum creatinine was measured by a Jaffe method (Modular P, Roche Diagnostic^®^, Mannheim, Germany), triglycerides and cholesterol levels were measured using the enzymatic method (Advia^®^ 1800 AutoAnalyzer, Germany) and the LDL-cholesterol fraction was calculated according to the Friedewald equation (19) when the plasma triglyceride level was below 400 mg/dL.

Blood pressure was measured with a digital sphygmomanometer Omron^®^ model HEM 705 CP by two consecutive measurements at a one-minute interval, with the individual seated after five minutes of rest. The diagnosis of hypertension considered the use of antihypertensive drugs or systolic blood pressure levels equal to or above 140 mmHg and diastolic levels equal to or above 90 mmHg (20).

Metabolic syndrome criteria consisted of the presence of three or more of the following components: waist circumference ≥ 94 cm in men and ≥ 80 cm in women; triglyceride levels > 150 mg/dL (1.7 mmol/L), HDL-cholesterol <40 mg/dL (1.0 mmol/L) in males and < 50 mg/dL (1.3 mmol/L) in females, systolic blood pressure ≥ 130 or diastolic blood pressure ≥ 85 mm Hg or treatment for previously diagnosed hypertension (21). Since the sample includes subjects with T1D, all patients fulfilled the condition of fasting plasma glucose > 100 mg/dL (5.6 mmol/L).

Individuals with values of GFR < 60 mL/min/1.73 m^2^ and/or albuminuria > 30 mg/g (19), at least two confirmatory measures, were diagnosed with diabetic kidney disease. The glomerular filtration rate (GFR) was calculated by the Chronic Kidney Disease Epidemiology Collaboration (CKD-EPI) (22) equation, considering gender and race.

The IR was determined by the Estimated Glucose Disposal Rate (EGDR) equation (23), which includes values of the waist-to-hip circumference (WHR), the presence of hypertension (considering one if present and zero if absent), and HbA1c:


$$\begin{array}{l} \mathrm{EGDR}\;(\text{mg}\cdot {\text{kg}}^{-1}\cdot {\text{min}}^{-1})=24.31-12.22\;(\text{WHR})-\\ 3.29\;(\text{Hypertension})-0.57\;(\text{HbAlc}) \end{array}$$

### Anthropometric evaluation and body adiposity markers

The interviewers received training to perform all anthropometrics measurements. Body weight and height were measured using a digital anthropometric scale Marte^®^ LS200A with a maximum capacity of 201 kg, and sensitivity of 50 g, with individuals barefoot and wearing light clothing. WC was measured using a metric tape of non-elastic material in the standing position, midway between the lowest costal rib and the iliac crest and hip circumference was measured at the largest circumference between the iliac crest and the trochanter (24).

The body adiposity markers WHtR, CI, LAP, BMI and BAI were calculated using the following equations:

1. **Waist-Height Ratio (WHtR)** (25): WC (cm)/Height (cm)
2. **Conicity Index (CI)** (26): $\frac{\text{WC }(\text{cm})}{0.109\surd \text{Body weight }(\text{kg})/\text{Height }(\text{m})}$
3. **Lipid Accumulation Product (LAP)** (27): $\begin{array}{l} {}[\text{WC }(\text{cm})-65]\times \text{serum triglycerides}\\ (\text{mmol}/\text{L})\text{ in men and }[\text{WC}(\text{cm})-58]\times \text{serum triglycerides }(\text{mmol}/\text{L})\text{ in women} \end{array}$
4. **Body Mass Index (BMI)** (28): $\text{Body weight }(\text{kg})/{\text{Height}(\text{m}}^{2})$
5. **Body Adiposity Index (BAI)** (29): $\frac{\text{Hip Circumference }(\text{cm})-18}{\text{Height }(\text{m})\surd \text{Height }(\text{m})}$

### Statistical analysis

One-way ANOVA was used to analyze parametric variables between EGDR groups. The Bonferroni test was used for post-hoc analysis. The Spearman correlation coefficient and Kruskal-Wallis test were used for nonparametric variables. The chi-square test was performed when appropriate, for categorical variables. The EGDR was analyzed in tertile since there is no cutoff value described in the literature. The groups were defined as tertile 1 ≤ 5.4; tertile 2 >5.4 and <8.4; and tertile 3 ≥ 8.4 mg.kg^−1^.min^−1^; the interpretation of results was based on the inverse relationship between EGDR and IR.

The areas under the curve (AUC) of receiver operating characteristics (ROCs) were calculated to measure the ability of adiposity markers to discriminate IR risk, considering the most insulin resistant individuals (EGDR ≤ 5.4 mg.kg^−1^.min^−1^). The cutoff values of body adiposity markers were defined according to values validated in the literature as WC ≥ 94 cm for men and ≥ 80 cm for women (21), WHtR > 0.5 (24) and BMI > 24.9 kg/m^2^ (27). The cutoff values of CI and LAP were determined according to analysis of sensitivity and specificity and 95% confidence interval, which were 1.19 for men and 1.15 for women and 16.1 for men and 18.1 for women, respectively. AUC analyses were assessed using the WinPepi Program version 11.47 and comparisons between AUCs were performed using the MedCalc Statistical Software version 16.4.

Poisson regression models were constructed to estimate the odds ratio (OR) and 95% confidence interval of associations between adiposity markers and IR. Each adiposity marker was analyzed individually, using the most insulin resistant group (tertile 1 of EGDR) as the dependent variable. Model 1 was adjusted for age, gender, serum triglycerides and glomerular filtration rate, while model 2 was adjusted for age, gender, serum triglycerides and the presence of diabetic kidney disease. The LAP was not adjusted for triglycerides in either model since it is contained in its equation.

All data were analyzed using Statistical Package for Social Sciences Software, version 18.0 (SPSS Inc., Chicago, IL, USA), considering a statistical significance of p < 0.05.

## RESULTS

A total of 128 subjects were included (51.5% women) with a mean age of 38.7 ± 11.3 years and median EGDR of 7.2 (4.4-8.7) mg.kg^−1^.min^−1^. The subjects were classified according to IR (stratified in EGDR tertiles) about clinical and laboratory characteristics (Table 1). Individuals with increased IR (lowest EGDR values) showed higher HbA1c and triglycerides, a higher prevalence of metabolic syndrome and the presence of diabetic kidney disease, and a lower GFR than individuals in the other groups. These individuals who were more resistant to insulin action also displayed higher values of WC, CI, WHtR and LAP when compared with individuals of the other tertiles (p < 0.05 for all) (Table 1). BMI shows borderline significance (p = 0.052), and there was no statistical difference for BAI (p = 0.975).

**Table 1:** Clinical and laboratory characteristics according to EGDR tertiles in subjects with type 1 diabetes

Characteristics	Total	1° tertile EGDR (≤5.4)	2° tertile EGDR (>5.4 <8.4)	3° tertile EGDR (≥8.4)	p-value
N	128	42	43	43	-
White skin color* N (%)	112 (87)	32 (76)	39 (91)	41 (95)	0.077
Age (years)	39 (30-49)	39.5 (28.7-52)	40 (30.5-50.0)	40 (30-46)	0.429
Women N (%)	66 (52)	20 (30)	22 (33)	24 (37)	0.750
Diabetes duration (years)	17.7 ± 8.9	19.8 ± 8.5	15.6 ± 8.6	17.6 ± 9.4	0.640
Glucose (mg/dL)	167.0 (122-286)	166.0 (32-513)	167.0 (42-699)	174 (38-442)	0.867
HbA1c (%)	8.7 (7.9-10.3)	10.3 (7-19.3)^b^	9.3 (6.1-12.2)^c^	8 (5.8-10)	< 0.001
Total cholesterol (mg/dL)	190.5 ± 37.5	198.6 ± 40.8	192.4 ± 35.0	180.9 ± 35.2	0.090
LDL-cholesterol (mg/dL)	108.2 (88.0-132.4)	109.7 (101.4-148.2)	101.8 (78.1-129.8)	101.8 (85.4-126)	0.114
HDL-cholesterol (mg/dL)	59.5 ± 17.4	37.3 ± 16	61.6 ± 19.9	59.7 ± 16.2	0.532
Triglycerides (mg/dL)	85 (60-118)	106.5 (27-218)^b^	84 (34-273)	78 (40-223)	0.040
Glomerular filtration rate (mL/min/1.73 m^2^)	97.6 ± 24.9	86.4 ± 29.7^a^^b^	101.4 ± 24	104.9 ± 15.7	0.001
Systolic blood pressure (mmHg)	123 ± 20	130 ± 24^b^	119 ± 20	118 ± 13	< 0.001
Diastolic blood pressure (mmHg)	75 ± 12	79 ± 11^a^	72 ± 14	73 ± 9	0.013
Presence of hypertension	51 (39.9)	40 (31.3)	11 (8.6)	0 (0)	0.064
Presence of diabetic kidney disease	46 (36)	24 (18.8)^a^,^b^	16 (11.7)	7 (5.5)	< 0.001
Presence of metabolic syndrome	41 (32)	28 (21.9)^a^,^b^	9 (7)	4 (3.1)	< 0.001
Waist circumference (cm)	82.8 ± 9.5	86.9 ± 9.0^b^	82.2 ± 9.5	79.5 ± 8.6	0.001
Waist-to-height ratio	0.49 ± 0.05	0.51 ± 0.47^a^,^b^	0.49 ± 0.54	0.47 ± 0.52	< 0.001
Conicity index	1.18 ± 0.07	1.21 ± 0.08^b^	1.17 ± 0.06	1.15 ± 0.06	0.001
Lipid accumulation product	19.5 (11.9-30.2)	25.7 (18.1-38.3)^b^	15.3 (10.4-30.9)	15.9 (8.8-20.8)	0.005
Body mass index (kg/m^2^)	24.6 ± 3.8	25.7 ± 4.2	24.5 ± 3.5	23.7 ± 3.6	0.052
Body adiposity index	58.3 ± 6.3	58.5 ± 5.2	58.3 ± 7.4	58.2 ± 6.2	0.975

EGDR: Estimated Glucose Disposal Rate (mg.kg^−1^.min^−1^).

* Ethnicity was self-reported as white or non-white. Data expressed in mean ± SD, median (q1-q3) and number of individuals (n) and percentage (%).

a Difference between tertile 1 and tertile 2.

b Difference between tertile 1 and tertile 3.

c Difference between tertile 2 and tertile 3. One-way ANOVA test (Bonferroni post hoc) or Kruskal-Wallis test as appropriated.

The correlation analyses between the adiposity markers and EGDR demonstrated a negative correlation for all analyzed variables: WC (*r* = −0.36; p < 0.01), WHtR (*r* = −0.39; p < 0.01), CI (*r* = −0.44; p < 0.01), LAP (*r* = −0.41; p < 0.01), BMI (*r* = −0.24; p < 0.01) and BAI (*r* = −0.08; p = 0.36).

Table 2 shows the description of body adiposity markers and their cutoff values in association with IR. After regression analysis adjusted for potential confounders, the variables WC, WHtR, CI, LAP and BMI remained associated with EGDR, as described in Table 3.

**Table 2:** Description of body adiposity markers to identify association with insulin resistance

Markers		AUC ± SE (CI 95%)	Cutoff value	Sensitivity% (CI 95%)	Specificity% (CI 95%)
**Waist Circumference (WC)**					
All patients (n = 128)		0.68 ± 0.05 (0.58-0.78)	-	-	-
	Women (n = 66)	0.62 ± 0.07 (0.48-0.77)	80.0	65.0 (43.3-81.0)	54.7 (39.9–68.8)
	Men (n = 62)	0.76 ± 0.06 (0.61-0.86)	94.0	45.5 (26.9-65.3)	85.0 (70.9–92.9)
**Waist-Height Ratio (WHtR)**					
All patients (n = 128)		0.70 ± 0.05 (0.60-0.79)	0.5	71.4 (56.43-82.8)	65.8 (55.3–75.1)
	Women (n = 66)	0.66 ± 0.07 (0.52-0.80)	0.5	70.0 (48.1-85.5)	68.9 (54.3–80.5)
	Men (n = 62)	0.74 ± 0.06 (0.61-0.86)	0.5	72.7 (51.8-86.9)	62.5 (47.0–75.8)
**Conicity Index (CI)**					
All patients (n = 128)		0.72 ± 0.05 (0.62-0.82)	1.19	78.6 (64.1-88.3)	52.9 (42.4–63.2)
	Women (n = 66)	0.64 ± 0.08 (0.48-0.80)	1.15	70.0 (48.1-85.4)	44.4 (30.9–58.8)
	Men (n = 62)	0.79 ± 0.06 (0.68-0.90)	1.19	86.3 (66.6-95.2)	62.5 (47.0–75.8)
**Lipid Accumulation Product (LAP)**					
All patients (n = 128)		0.70 ± 0.05 (0.61-0.79)	13.8	85.7 (72.1-93.3)	41.2 (31.3–51.8)
	Women (n = 66)	0.62 ± 0.07 (0.48-0.77)	18.1	70.0 (48.1-85.4)	48.9 (34.9–63.0)
	Men (n = 62)	0.80 ± 0.05 (0.69-0.91)	16.1	81.8 (61.5-92.7)	62.5 (47.0–75.8)
**Body Mass Index (BMI)**					
All patients (n = 128)		0.62 ± 0.05 (0.51-0.72)	24.9	59.5 (44.5-72.9)	62.3 (51.7–71.9)
	Women (n = 66)	0.58 ± 0.08 (0.43-0.74)	24.9	50.0 (29.9-70.1)	64.4 (49.8–76.8)
	Men (n = 62)	0.65 ± 0.07 (0.50-0.79)	24.9	68.2 (47.3-83.6)	60.0 (44.6–73.6)

This analysis included individuals more insulin resistant (tertile 1). The cutoff values of WC, BMI and WHtR were based on values already described in the literature and cutoff values of CI and LAP were chosen according to sensibility and specificity analysis. AUC: area under the curve. Comparisons between AUC (all patients) of body adiposity markers: WC vs. WHtR (p = 0.50), WC vs. CI (p = 0.21), WC vs. LAP (p = 0.63), WC vs. BMI (p = 0.09), WHtR vs. CI (p = 0.62), WHtR vs. LAP (p = 0.87), WHtR vs. BMI (p < 0.01), CI vs. LAP (p = 0.74), CI vs. BMI (p = 0.09), and LAP vs. BMI (p = 0.07).

**Table 3:** Poisson regression model to determine the association between body adiposity markers (according to each cutoff value) and insulin resistance (EGDR ≤ 5.4 mg.kg^−1^.min^−1^) in individuals with type 1 diabetes (n = 128)

Variables	OR	CI (95%)	p-value
**MODEL 1**			
Waist Circumference	2.07	1.27-3.37	0.003
Waist-Height Ratio	2.77	1.59-4.79	<0.001
Conicity Index	2.59	1.43-4.66	0.002
Lipid Accumulation Product	2.27	1.25-4.11	0.007
Body Mass Index	1.78	1.09-2.91	0.019
**MODEL 2**			
Waist Circumference	1.78	1.09-2.90	0.019
Waist-Height Ratio	2.44	1.41-4.22	0.001
Conicity Index	2.17	1.18-3.99	0.013
Lipid Accumulation Product	2.15	1.18-3.93	0.012
Body Mass Index	1.62	1.03-2.57	0.036

EGDR: Estimated Glucose Disposal Rate (mg.kg^−1^.min^−1^). OR: odds ratio. CI: confidence interval. All models were analyzed individually for each body adiposity marker. The cutoff values of markers were waist circumference 94 for males and 80 for females, WHtR 0.5, conicity index 1.19 for males or 1.15 for females, lipid accumulation product 16.1 for males or 18.1 for females and body mass index 24.9. Model 1: Adjusting for age, gender, triglyceride and glomerular filtration rate. Model 2: Adjusting for age, gender, triglyceride and presence of diabetes kidney disease. The lipid accumulation product was not adjusted for triglycerides since is contained in its equation.

## DISCUSSION

In the present study, most of the adiposity markers were positively associated with IR, except for BAI. The WHtR and CI markers had the best association with IR defined according to EGDR. Previous studies (14,30) demonstrated that WHtR helps to discriminate metabolic syndrome in a sample of Italian adolescents (14) and in adults with T1D (30). Also, WHtR seems to be the best anthropometric measure to estimate visceral fat in T1D, independent of albuminuria stage and sex (31).

There are few articles in the literature that have evaluated adiposity markers associated with IR in individuals with T1D. Nevertheless, several papers (14,30-32) found the same direction for the association of WHtR with metabolic syndrome and visceral obesity, and its complications have already been demonstrated to be a risk factor for the development of IR (1,2,5,8,33).

Regarding cutoff value, Ashwell and cols. (34) showed that a boundary value of 0.5 for WHtR indicates an increased morbidity and mortality risk for men and women in different ethnic groups and has been proven to be more sensitive than BMI. In a cross-sectional study (33) that evaluated men and women, the AUC of WHtR was significantly greater than BMI and WC to predict diabetes, hypertension, high total cholesterol, high triglycerides and low HDL-cholesterol (p < 0.05 for all) in both genders. In our study, the ROC curve analysis showed that all markers had a similar AUC to discriminate IR; however, WHtR had the best association with IR.

WC is a simple and non-invasive method to apply in various equations to identify central obesity and IR risk. However, the isolated measure of WC does not take height into account; hence it can classify individuals with the same WC and different heights as equal risks. In the present study, when we incorporated height and WC in the same equation, it proved to be a better parameter for the association of IR in adults with T1D.

In addition, the visceral obesity evaluation in healthy subjects (35) demonstrated that CI was one of the most accurate markers with which to determine visceral obesity, especially in men, even compared to WHtR, which was better than CI for older women only. In our study, CI and WHtR were suitable parameters for the identification of individuals with a higher risk of IR in both genders. They had the best sensitivity, but it was highlighted that the specificity of CI was low for women. There is a difference between fat distribution according to gender. Usually, men have higher visceral adipose tissue than women, despite factors such as age and visceral adiposity being strongly associated with cardiometabolic risk in women (36).

A Brazilian cross-sectional study (35) demonstrated that CI was the most accurate marker with which to assess visceral obesity, especially in adults and elderly healthy men. Furthermore, when evaluated in patients with T2D (15), CI is a good indicator of a high risk of coronary heart disease (61.9 ± 9.5 years) using 1.35 as a cutoff value (p < 0.039). In our study, CI was a good measurement with which to identify IR in patients with T1D using a cutoff value of 1.15 for women (sensitivity: 70.0% and specificity: 44.4%) and 1.19 for men (sensitivity: 86.3% and specificity: 62.5%). In this study of Brazilians (15), the cutoff value of CI was higher in T2D than in our study of T1D, reinforcing the difference in body adiposity between these populations. Another factor that could be attributed is the age difference of the samples (61.9 ± 9.5 years vs. 38.7 ± 11.3 respectively), as body composition naturally changes throughout the years, especially in the elderly population.

Due to the trend of weight gain in T1D, using CI may be a way to discriminate alterations in metabolism, such as RI. This is an essential finding as CI evaluates central obesity and can detect changes in body fat distribution with a simple measurement method, allowing comparisons between individuals with different body weight and height dimensions and an increased health risk.

In addition, LAP was demonstrated to be most accurate for the detection of IR compared to BMI, WC and WHR in women with polycystic ovary syndrome compared to healthy women (p < 0001) (37). In the current study, LAP analysis was also demonstrated to be significant for IR identification. This measure is an interesting parameter since it includes waist circumference and triglycerides in its equations, and both measures are factors used to classify metabolic syndrome. Individuals with T1D and metabolic syndrome exhibit higher visceral adiposity compared with healthy subjects (38). A recent study (38) evaluated LAP, VAI and the triglyceride/HDL-cholesterol ratio; both were associated with metabolic syndrome in patients with T1D. The LAP cutoff value of this study was 27.57 (AUC = 0.842, sensitivity 80.0%, specificity 74.0%). They presented a higher LAP value than our study, probably due to the higher average waist circumference and different body composition assigned to ethnicity in their sample of T1D.

Although many studies show that BMI is a good parameter with which to measure obesity, it was not the best parameter in the population with DM1. In the present study, BMI was not shown to be the best measure of IR in individuals with T1D, probably because it evaluates the body weight to height ratio, and does not consider the amount of abdominal adipose tissue or the body muscle composition. Furthermore, BAI was not associated with IR and T1D. A cohort study (39) of 698 Mexicans indicated that BAI could be considered a global adiposity measure; however, it did not prove better than BMI for identifying adults who are at cardiovascular risk. BAI considers hip circumference and height in its equation; our study showed that equations that include WC had a better association with IR in subjects with T1D.

The limitations of this study were inherent to a cross-sectional design. Thus, it was not possible to determine a cause-and-effect relation, but it was possible to report associations. Also, it is important to recognize that these adiposity markers are indicators of risk and not diagnostic of IR. The present study was conducted in a specific population with T1D coming from a university hospital, and studies that evaluated the cutoff values of adiposity markers were conducted with different populations, making it difficult to compare with subjects with T1D.

In summary, this study reports that the body adiposity markers investigated are suitable parameters to be assessed in patients with T1D, especially IC and WHtR. Therefore, the authors suggest that health professionals can use these markers as routine, as they are simple to employ in clinical practice, and have a good association with IR in patients with T1D.
